# The anthropogenic consequences of energy consumption in the presence of uncertainties and complexities: evidence from World Bank income clusters

**DOI:** 10.1007/s11356-021-17476-5

**Published:** 2021-11-20

**Authors:** Festus Fatai Adedoyin, Elma Satrovic, Maureen Njideka Kehinde

**Affiliations:** 1 Department of Computing and Informatics, Bournemouth University, Poole, UK; 2 Department of Economics, University of Novi Pazar, Novi Pazar, Serbia; 3 Department of People and Organizations, Bournemouth University, Poole, UK

**Keywords:** Emissions, Energy consumption, Uncertainties, Complexities, World Bank Income Clusters

## Abstract

In environmental management, many studies have examined the energy consumption-emission nexus in detail. However, for the first time in the literature, this study considers how the Economic Complexity Index (ECI) and economic policy uncertainty (EPU) moderate the contribution of energy consumption to emissions for the four World Bank Income clusters. The system generalised methods of moments are applied to data for 109 countries from 1996 to 2016. Based on the main model (grouped clusters) estimations, the result revealed the existence of the environmental Kuznets curve (EKC) hypothesis. Also, an increase in air transport and consumption of energy releases more carbon emissions to the climate. Interestingly, ECI decreases carbon emission significantly while EPU does not have a significant impact. Moreover, the study revealed that ECI moderated the impact of other variables on emission, but EPU is not a significant moderator. Furthermore, a comparative analysis among the four incomes suggests that the EKC hypothesis holds only in the high-income clusters; ECI is a significant predictor of carbon emission in the four clusters, but it only decreases the emission in high-income clusters. This corroborates the debate on climate change and the productive capacity of high-income countries. Given the foregoing, several policy measures were recommended.

## Introduction

In today’s world, climate change is arguably the most severe challenge of our time with long-term serious impacts for a sustainable low carbon future of our planet. Climate change due to global warming is one of the defining issues of our time. The main cause of climate change is the greenhouse effect (Satrovic, 2019). When burnt, fossil fuels release carbon dioxide and other greenhouse gases into the air, causing the planet to heat up. In other words, human activity is the main cause of climate change. More and more attention from all over the world has been paid to the issue of how to reduce carbon emissions and mitigate climate change. Clarifying the influence of the factors behind the growth of greenhouse gas emissions is significant for efforts to meet its challenge. Therefore, this study examines the determinants of carbon emissions, introducing the level of economic complexity and economic policy uncertainty.

There is an extensive body of literature that investigated the carbon emission-economic growth link (Boleti et al, 2021; Adedoyin et al., 2021; Khan et al., 2021; Can and Gozgor, 2017; Satrovic and Dağ, 2019; Doğan et al., 2019; Tariq et al., 2017; Alola, 2019; Anser et al., 2021a; Mehmood, 2021; Khan and Hou, 2021; Adedoyin et al., 2020; Muslija et al., 2020; Murshed et al., 2020; Ahmad et al., 2021; Chandio et al., 2020; Bese et al., 2020; Verbič et al., 2021; Satrovic and Muslija, 2019; Mujtaba and Jena, 2021; Adebayo, 2020). The environmental Kuznets curve (EKC) hypothesis was developed by Grossman and Krueger (1991, 1995) to emphasise this nexus. Corresponding to the Kuznets curve of inequality (Kuznets, 1955) developed by Simon Kuznets, the EKC also proposed an inverted-U hypothesis. Grossman and Krueger (1991, 1995) anticipated that economic growth increases ecological impediments until at a certain point. In other words, environmental degradation rises in the early stages of economic development but falls in the latter stages.

Anxieties about economic policy uncertainty (EPU) related to economic decisions are higher than ever before in today’s interconnected world. These anxieties have escalated across the globe causing global political and economic uncertainty. In addition, the reports of the International Monetary Fund (Ahir et al., 2021) suggest that the economic growth of the USA and the European Union is a key driver of economic activity around the world. This is also true when it comes to global uncertainty. Technological change and globalisation have changed the quality of life leading to a higher level of uncertainty than ever before. Consequently, economic and political policy volatilities have proliferated globally. Global uncertainties (political, economic, social, health or war) are likely to influence economic activity (Adams et al., 2020). For instance, a health issue due to the COVID-19 pandemic has brought economic activity to a near-standstill.

World economic activity recorded one of the fastest falls in history. Overall, EPU influences the business/industry environment and this, in turn, affects the process of making decisions in business entities. Herein, Bloom (2009) together with Baker et al. (2016) inspired the studies that investigate the economic effects of economic policy uncertainty (Yu et al., 2020; Chu and Le, 2021; Zakari et al., 2021; Pirgaip and Dincergok, 2020; Wang et al., 2020). EPU may also have environmental effects. For instance, EPU may prompt economic entities to use classical and environmentally harmful production methods generating strong carbon emissions. Moreover, EPU influences consumption and investments, which in turn hinders environmental degradation. Environmental degradation can be also improved due to renewable energy consumption, innovations or research and development (Anser et al., 2021b).

**Fig. 1:**
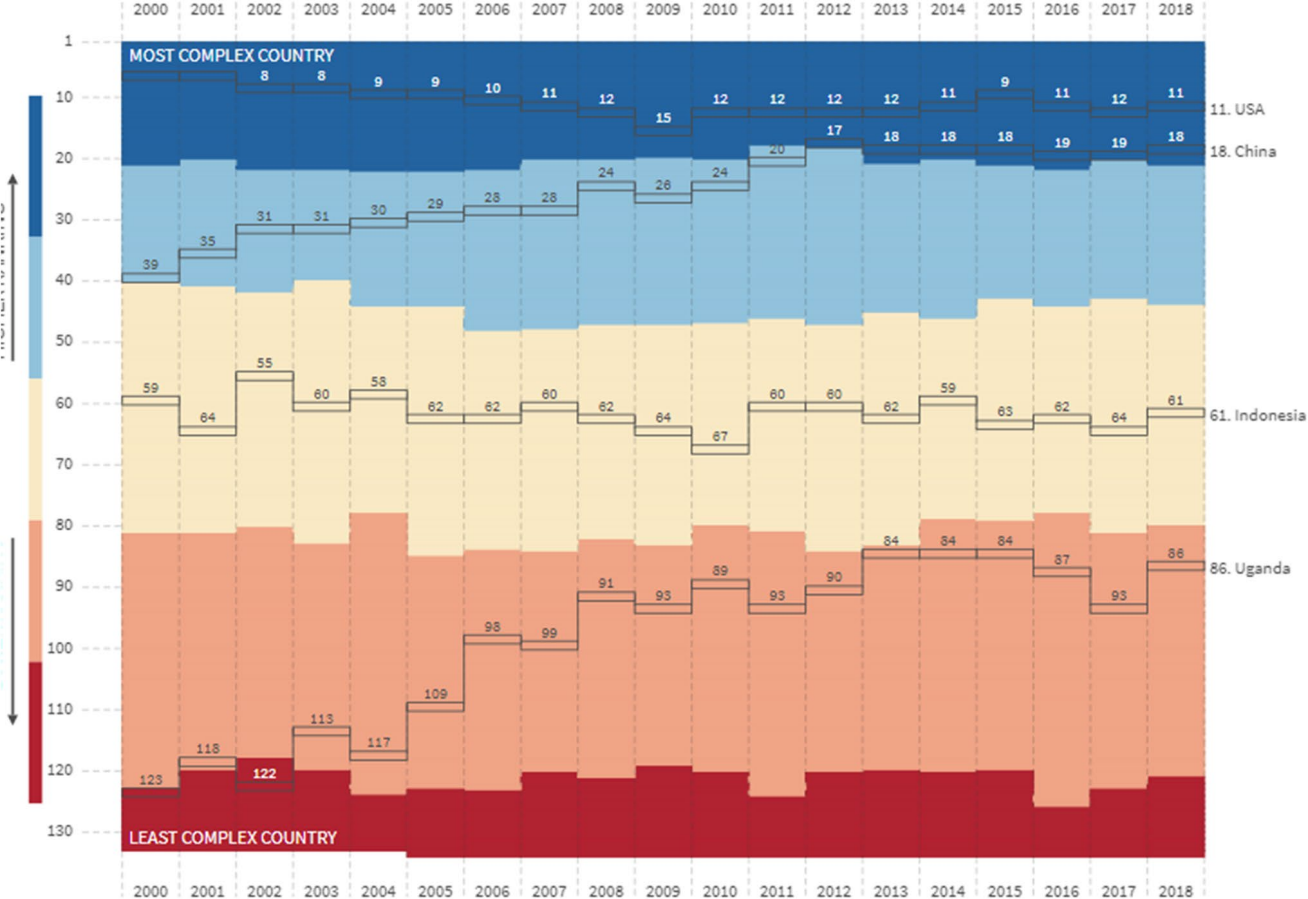
Country complexity ranking

The interconnected global economy resulted in increased complexity. The breaking study of Hidalgo and Hausmann (2009) served to introduce the Economic Complexity Index (ECI). In brief, it is a combination of a country’s productive output. Hausmann et al. (2014) alternatively define economic complexity as a country’s ability to produce high value-added products and their productivity. A novel measure known as the ECI is firstly introduced by Hidalgo and Hausmann (2009) to infer information about countries’ productive capabilities from their export baskets (Can and Gozgor, 2017; Yilanci and Pata, 2020). Countries with high product diversification have a more complex economy and are likely to have higher productivity (Fig. 1). On the contrary, simpler products are produced in simpler economies and these are likely to have lower productivity. Herein, ECI is commonly used as a proxy for the progress in an economy (Yilanci and Pata, 2020). As such, ECI also relates to carbon emissions and environmental degradation. Yilanci and Pata (2020) suggest that simpler economies generally focus on simpler products (agricultural, raw minerals) and thus are unlikely to cause severe environmental damages. On the contrary, more developed countries focus on more complex and diversified products causing severe environmental damages.

Furthermore, empirical studies aimed to validate the EKC hypothesis incorporating various determinants of carbon emissions such as transportation sector and energy (electricity) consumption. The transportation sector is an important component of the economy and a common tool used for development. However, this sector largely increases carbon emissions (Erdogan et al., 2020; Nassani et al., 2017). IPCC (2014) breaks down the global greenhouse gas emissions by the economic activities and reports that transportation is responsible for 14% of 2010 global greenhouse gas emissions. In addition, ATAG (2019) reports that the global aviation industry produces around 2% of all human-induced carbon emissions while aviation is responsible for 12% of carbon emissions from all transport sources, compared to 74% from road transport. However, aircraft emissions are more dangerous than transport emissions that come from road vehicles because carbon emissions from the airline industry are released high in the atmosphere and have potent atmospheric effects that heat the planet.

Flights are energy-intensive and depend on fossil fuels. Transportation and energy consumption are mutually interdependent with each other. Energy consumption may increase carbon emissions while transportation contributes to the energy-environmental degradation nexus (Satrovic and Abul, 2020). The intensive use of trains offers eco-friendly transportation methods and aims to reduce the travel cost and support environmental protection. Electric trains have always had no direct carbon emissions. However, IPCC (2014) reports that electricity and heat production are responsible for 25% of 2010 global greenhouse gas emissions. Specifically, energy (electricity consumption) along with economic performance can influence carbon dioxide emissions.

Global uncertainties and especially the COVID-19 pandemic that has resulted in the downturn of economic activity in many countries indicated that economic growth in the key systemic economies (e.g., the USA and European Union) determines economic activity in the rest of the world. However, there are limited studies on the question of whether or not uncertainty in systemic economies matters for uncertainty in the rest of the world. The objective of the current study is to investigate the air transport emissions nexus within the EKC framework and to explore whether the roles of economic policy uncertainty and economic complexity will matter. Yet, to the best of our knowledge, no study has investigated the effect of economic policy uncertainty and economic complexity on carbon emissions in the context of World Bank income clusters.

The “Literature review” provides a brief overview of the literature. “Data and methods” presents data and methodology used. The “Results and discussions” contains empirical results and discussions. In section “Conclusion and policy implications”, we propose vital directions for policymakers.

## Literature review

### Economic Complexity Index and environment nexus

The impact of economic complexity on the environment remains relatively unexplored. Limited studies are identifying the economic complexity-environment nexus, and most of them use carbon emissions as a proxy for environmental performance (Table 1). Kaufmann et al. (1998) outlined that the time component and the composition of economic activity influence environmental quality. Countries with high (low) economic complexity tend to specialise in high (low) product complexity index products (Mealy et al., 2019). High economic complexity imposes a shift from low-productivity agricultural products to higher-productivity sophisticated products. A transition from low- to higher-productivity sectors rises in demand for energy, which in turn increases the carbon emission and hampers environmental quality. Contrastingly, ECI reveals the transformation of the economic structure and looks to explain the knowledge accumulated in a population and state-of-the-art production processes (Boleti et al., 2021). This study analysed the association between ECI and ecological impediments in the period of 2002–2012 using the data for 88 developed and developing countries. The export sophistication is not found to have a negative environmental effect. Nevertheless, economic complexity negatively influences air quality.

**Table 1:** Interactions between environment, economic growth, energy-related or macroeconomic variables

Author(s)	Sample-period	Method	Energy-macroeconomic variables
Boleti et al. (2021)	88 countries, 2002–2012	POLS	ECI ( +), population ( −), agriculture ( −), industry ( −), corruption ( +), trade ( +), urban ( +), education ( −)
Can and Gozgor (2017)	France, 1964–2014	DOLS	EC ( +), ECI ( −)
Neagu (2019)	25 countries, 1995–2017	FMOLS, DOLS	Energy intensity ( +), ECI ( +)
Chu (2020)	118 countries, 2002–2014	GMM	Institutions ( −), gross fixed capital formation ( +), industry value added ( +), renewable energy ( −), ECI ( +)
Azizi et al. (2019)	99 countries, 1992–2017	DOLS	ECI ( −)
Neagu and Teodoru (2019)	25 countries, 1995–2016	DOLS, FMOLS	EC ( +), ECI ( +)
Doğan et al. (2019)	55 countries, 1971–2014	QE	ECI ( −), EC ( −), trade ( −), urban ( +)
Neagu (2020)	48 countries, 1995–2014	DOLS, FMOLS	EC ( +), ECI ( +)
Yilanci and Pata (2020)	China, 1965–2016	FARDL	EC ( +), ECI ( +)
Anser et al. (2021)	10 countries, 1990–2015	PMG-ARDL	EC ( +), EPU ( +), urban ( +)
Adedoyin et al. (2020)	10 countries, 1995–2015	FMOLS, DOLS	EC ( +), EPU ( +), tourism ( +)
Adams et al. (2020)	10 countries, 1996–2017	PMG/MG-ARDL	EC ( +), EPU ( +), geopolitical risks ( −)
Chu and Le (2021)	7 countries, 1997–2015	FMOLS	EC ( +), ECI ( +), EPU ( −), renewable energy ( −)
Zakari et al. (2021)	22 countries, 1985–2017	PMG-ARDL	EC ( +), EPU ( +), renewable energy ( −)
Pirgaip and Dincergok (2020)	7 countries, 1998–2018	GCT	Unidirectional causality: EPU to EC in Japan; EPU to CO_2_ in the USA and Germany
Wang et al. (2020)	United States, 1960–2016	ARDL	EPU ( +), energy prices ( −)
Jiang et al. (2019	United States, 1985–2017	GCT	CO2 is affected by EPU
Yu et al. (2020)	China, 2008–2011	IVR	EC ( +), EPU ( +)

*GMM* generalised method of moments, *FARDL* fractional Fourier frequency autoregressive-distributed lag, *PMG-ARD*L pooled mean group autoregressive-distributed lag, *MG-ARDL* mean group autoregressive-distributed lag, *GCT* Granger causality tests, *IVR* instrumental variables regression, *DOLS* dynamic ordinary least square, *FMOLS* fully modified ordinary least square; *QE* quantile estimation, *POLS* pooled ordinary least squares, *ECI* Economic Complexity Index, *EC* energy consumption, *EPU* economic policy uncertainty

In another study, Can and Gozgor (2017) analysed the determinants of carbon emissions under the EKC framework. The authors investigated the case study of France from 1964 to 2014. Empirical findings showed that the EKC hypothesis exists in France. In addition, energy consumption is reported to be an important driver of carbon emissions. In addition, economic complexity reduces environmental degradation in the long run. Similarly, Neagu (2019) investigates the ECI-environmental pollution nexus in the inspected panel of 25 European Union countries. Using the timespan of 1995–2017, the author showed that the EKC phenomenon exists, meaning that export sophistication increases ecological impediments to a point where this trend is reversed. These findings also outline a statistically significant positive relationship between energy intensity and carbon emissions. Further elaborations of the concept of the EKC phenomenon are presented by Chu (2020). For econometric analysis, the author has prepared a dataset of 118 countries. Empirical results revealed the positive association between ECI and climate change to a point where this trend is reversed. In other words, a shift from low- to higher-productivity sectors does not necessarily improve the environmental quality. In addition, this study consistently confirms the validity of the EKC hypothesis.

Parallel to this, Azizi et al. (2019) used the data from 99 countries to analyse the validity of the inverted-U-shaped relationship between economic activity and carbon emissions in the period of 1992–2017. The findings of this paper suggest the validity of the EKC hypothesis in the context of 99 analysed countries. Moreover, economic complexity reduces environmental depletion. Using the case study of Lancang-Mekong Cooperation countries, Liu et al. (2021) analyse the relationship between energy consumption and economic complexity under the context of a sustainable environment. Annual panel data are collected from 1991 to 2017. The findings of this paper clearly outline unidirectional linkage amid energy use and ECI. Neagu and Teodoru (2019) use the panel of European Union countries to explore the association between environmental quality, energy consumption, and economic complexity. The results of this study suggest a long-run association between selected macroeconomic variables. In addition, energy consumption and economic panels have a statistically significant impact on greenhouse gas emission. Accordingly, the authors highlight the necessity to consider economic complexity while determining macroeconomic and energy policies. Doğan et al. (2019) analyse the economic complexity-environment nexus for 55 countries. Using the period of 1971–2014, this study explores the validity of the EKC hypothesis. The results clearly outline an important role of economic complexity in environmental depletion. Herein, the world’s economies assigned to three income groups should modify manufacturing-related policies to promote sustainable development. The selected countries demonstrated the EKC evidence.

Instead of using carbon dioxide emissions, Neagu (2020) and Yilanci and Pata (2020) proposed the ecological footprint as a proxy for environmental degradation. The case study of 48 economies was analysed by Neagu (2020) from 1995 to 2014. A positive long-run association between fossil fuel energy consumption, economic activity, ECI and ecological footprint is revealed in the inspected sample. Herein, the selected macroeconomic variables should be seen as a threat to environmental quality. Similarly, Yilanci and Pata (2020) used the case study of China to test the validity of the EKC hypothesis in the period 1965–2016. Energy consumption and ecological complexity have a statistically significant positive impact on ecological footprint. It is also worth mentioning that the EKC hypothesis is not valid for China in the observed period. Overall, the shift in economic activity from low to high productivity is not affecting in alleviating environmental issues in China. Based on these findings, it can be easily concluded that Chinese exported products are environmentally unfriendly. Considering our discussion so far, the comparison across country export baskets enables us to depict countries’ productive capabilities. Countries that have higher rankings on the Economic Complexity Index (more complex countries) are exporting complex products and consequently report higher GDP per capita (Mealy and Teytelboym, 2020). Hence, more complex countries offer better conditions for developing more technologically sophisticated products that benefit the environment.

### Economic policy uncertainty and environment nexus

Complexity is an important determinant of economic policy uncertainty. However, Baker et al. (2016) revealed that innovation eases this complexity. To investigate the role of policy uncertainty, Baker et al. (2016) developed an index of economic policy uncertainty (EPU). In contrast to the uncertainty-growth studies, those on the uncertainty-environment nexus are scarce and emerging (Table 1). Anser et al. (2021b) are exploring the relationship between uncertainty and manmade emissions of carbon dioxide in the case of the top ten carbon emitter countries. Using the annual panel data from 1990 to 2015, the authors outline that economic policy uncertainty increases carbon emissions in the long run. Short-run effects however suggest a negative impact of economic policy uncertainty on carbon emissions. Herein, the selected top ten carbon emitter countries should enforce renewable energy, modern technologies, and innovation that will improve environmental quality.

In another study, Adedoyin et al. (2020) examined whether or not economic policy uncertainties matter into the nexus among energy, tourism, economic growth, and environmental depletion. The authors evidenced that EPI tourism and energy consumption are critical determinants of environmental depletion from 1995 to 2015. Herein, the authors outlined that sustainable growth is possible under the condition that environmental policies reduce the negative effects of economic activities. In that way, environmental policies induce environmental quality. Similarly, Liu et al. (2020) attempted to evaluate the impact of EPU on the tourism-growth nexus. Using the case study of Hong Kong, Chinese, and global EPU, the authors suggested that EPU influences the size of the impulse response. In addition, different levels of economic policy uncertainty have different impact intensities.

According to Adams et al. (2020), the feedback hypothesis between EPU and energy use is confirmed from 1996 to 2017. Using the case study of countries with high geopolitical risk, the authors reported a positive significant impact of energy consumption and economic activity on carbon emissions. The findings of this paper also suggest a bidirectional causal linkage amid environmental depletion and energy use, environmental depletion and EPU, environmental depletion and real GDP per capita. However, the authors showed the unidirectional causality running from environmental depletion to geopolitical risks. More recently, Chu and Le (2021) showed a cointegration relationship between economic complexity, EPU, energy collected from renewable sources, and energy intensity. The findings outlined the adverse environmental impact of high energy intensity whereas renewable energy and economic policy uncertainty improve environmental quality. The authors also confirmed the validity of the EKC hypothesis for G7 countries. Overall, this study suggests that government should reduce uncertainties around economic policies and support clean technologies and renewable energy sources that will be beneficial for the environment.

Furthermore, Zakari et al. (2021) analysed the nexus between economic policy uncertainty and the environment. The authors reported the negative environmental effects of energy consumption and EPU from 1985 to 2017 for 22 Organisation for Economic Co-operation and Development (OECD) economies. However, the findings reported that renewable energy improves environmental quality in the long run. The short-run findings outlined a direct impact of energy use and economic activity on carbon emissions. Besides, the authors reported a unidirectional causality running from economic activity to carbon emissions, and EPU to carbon emissions. The causal relationship between EPU, energy consumption and environmental degradation is investigated by Pirgaip and Dincergok (2020). On the panel of G7 countries, the authors revealed unidirectional causality running from EPU to energy EPU to carbon dioxide emission in the period of 1998–2018. The authors draw important policy implications recommending that G7 governments should consider the potential negative environmental impacts of EPU on energy conservation policies which should be promoting green energy and reducing carbon emissions.

The impacts of EPU on climate change were examined by Wang et al. (2020) for the USA in the period of 1960–2016. The authors showed that economic growth increases environmental degradation in the long run. Economic policy uncertainty is also found to drive anthropogenic emissions in the long run. However, the authors revealed a negative impact of energy prices on carbon emissions. In another study, Jiang et al. (2019) explored whether or not EPU matters for climate change. Using the US sector-level data, the authors reported that climate change is influenced by EPU. It is worth mentioning that there is evidence of a causal relationship between EPU and climate change only for the industry and commercial sectors. Similarly, Yu et al. (2020) use China’s provincial EPU index to analyse the linkage between EPU and firms’ greenhouse gas emissions. This paper suggests that China’s provincial EPU index increases greenhouse gas emissions at the firm level. Overall, the conclusion drawn from previous studies affirms that conservative policies proved most effective at times of high economic policy uncertainty (Al-Thaqeb and Algharabali, 2019). This is because high EPU increases borrowing costs reduces a firm’s capital expenditures and consequently causes an economic downturn.

### Research gap

Previous paragraphs helped us to identify a distinguishing pattern of characteristics in existing studies. First, the recent studies were analysing the validity of the EKC hypothesis by mainly focusing on the growth-environment nexus. Second, early studies controlled for energy-related or macroeconomic variables which are recognised as driving factors of carbon emissions, alongside economic growth. Third, there is limited evidence of whether or not air transport affects environmental pressure. Fourth, recent studies explore the direct impact of economic complexity and economic policy uncertainty on carbon emissions and do not take into consideration the moderating effects of both factors.

Although Khan et al. (2021) and Alola and Joshua (2020) are deepening the income-climate change narrative by capturing the World Bank come clusters, our study contributes to the existing literature in a few dimensions. First, the current study takes into consideration the direct effect of economic complexity and economic policy uncertainty while investigating the behaviour of the EKC phenomenon. Secondly, for the first time, the current study applies the economic complexity index and economic policy uncertainty as moderating variables on the overall model. After the moderators are added to the regression model, we interpret the results considering that variables in the models are now conditional on the level of economic complexity and economic policy uncertainty. Third, the current study introduces air transport as a proxy for the transport industry. The transport industry is introduced with a particular aim to unveil the impact of air transport on environmental pressure capturing the World Bank come clusters.

## Data and methods

### Data and variables

In this study, the factor that affects climate change in World Bank income clusters (Table 8 in the Appendix) for a period of 1996–2016 is examined. The dependent variable used is carbon emission per capita which is climate change, and the variables of interest for independent ones are real GDP per capita growth, squared real GDP per capita growth, energy use and air transport system. Furthermore, the main important interest in this study is the application of the economic complexity index and economic policy uncertainty as moderating variables on the overall model. The data gathered is panel data, and the variables of interest are presented in Table 2.

**Table 2:** Description of variables

Variables	Acronym	Data source
Moderation variable 1 (energy use × ECI)	LENUECI	Author calculation
Moderation variable 2 (energy use × EPU)	LENUEPU	Author calculation
Carbon dioxide emission per capita	CO2PC	World Bank Development Indicator
Real GDP per capita growth	RGDP	World Bank Development Indicator
Squared real GDP per capita growth	RGDP2	Author calculation
Energy use	EU	World Bank Development Indicator
Air transport	AIR	World Bank Development Indicator
Economic complexities Index	ECI	ATLAS of economic complexity index
Economic policy uncertainties	EPU	World Uncertainty Index

### Model and method

Subject to actualizing the purpose of this research, panel data of World Bank income clusters was pulled for a period ranging from 1996 to 2016; the exploratory data analysis of the variables was assessed using four measures of statistics (mean, standard deviation, minimum, and maximum) to check the behaviour and variation of the variables at the level and logged variables, correlation matrix, and scatter plot to pre-investigate the relationship between the dependent (LCO2PC), and the log of independent variables. Also, the multicollinearity test—the existence of association among independent variables—was diagnosed using the correlation matrix (Table 4); the result revealed that none of the correlation coefficients among the independent variables exceed the threshold of 70% indicating that the employed do not suffer from multicollinearity. After this, panel model techniques were set up. The models estimated the World Bank income clusters, and then across each cluster entity.

The models have pooled OLS or least square dummy variable (LSDV, Eq. 3) techniques, fixed effect (FE, Eq. 4) model techniques, random effect (RE, Eq. 5) model techniques and one-system GMM (Eq. 6). All these were employed to ascertain the robustness of the result. For instance, pooled OLS (or LSDV) can capture the income clusters as dummy variables while at the same time estimating normal OLS without considering the region-specific and/or time effect, FE can capture the actual effect of change in income clusters without considering the error components between among the clusters, and RE model can capture the how the income clusters and time influence the variation among the clusters (error component). However, none of them except one-system GMM could capture lagged dependent variables as another factor of the dependent variables. This is so because one-system GMM could eliminate the error of serial correlation or autocorrelation of the error term and the problem of heteroscedasticity.

1 $$\mathrm{CO}2\mathrm{PC}=\mathrm f(\mathrm{RGDP},\mathrm{RGDP}2,\mathrm{AIR},\mathrm{ECI},\mathrm{ENU},\mathrm{EPU})$$

2 $$\mathrm{LCO}2\mathrm{PC}=\mathrm f(\mathrm{LRGDP},\mathrm{LRGDP}2,\mathrm{LAIR},\mathrm{ECI},\mathrm{LENU},\mathrm{EPU})$$

3 $${\mathrm{lnCO}2\mathrm{PC}}_{\mathrm{it}}=\beta_{0\mathrm i}+\beta_{1\mathrm i}\mathrm{lnRGDP}+\beta_{2\mathrm i}\mathrm{lnRDGP}2+\beta_{3\mathrm i}\mathrm{lnAIR}+\beta_{4\mathrm i}\mathrm{ECI}+\beta_{5\mathrm i}\mathrm{lnENU}+\beta_{6\mathrm i}\mathrm{lnEPU}+\gamma_{\mathrm i}{\left(\mathrm{dummy}\right)}_{\mathrm n-1}{+\varepsilon}_{\mathrm i,\mathrm t}$$

4 $${\mathrm{lnCO}2\mathrm{PC}}_{\mathrm{it}}=\beta_{0\mathrm i}+\beta_{1\mathrm i}\mathrm{lnRGDP}+\beta_{2\mathrm i}\mathrm{lnRDGP}2+\beta_{3\mathrm i}\mathrm{lnAIR}+\beta_{4\mathrm i}\mathrm{ECI}+\beta_{5\mathrm i}\mathrm{lnENU}+\beta_{6\mathrm i}\mathrm{lnEPU}{+\mathrm u}_{\mathrm i,\mathrm t}$$

5 $${\mathrm{lnCO}2\mathrm{PC}}_{\mathrm{it}}=\beta_{0\mathrm i}+\beta_{1\mathrm i}\mathrm{lnRGDP}+{\beta}_{2i}\mathrm{lnRDGP}2+\beta_{3\mathrm i}\mathrm{lnAIR}+\beta_{4\mathrm i}\mathrm{ECI}+\beta_{5\mathrm i}\mathrm{lnENU}+\beta_{6\mathrm i}\mathrm{lnEPU}{+\mathrm u}_{\mathrm i,\mathrm t}{+\varepsilon}_{\mathrm i,\mathrm t}$$

6 $${\mathrm{lnCO}2\mathrm{PC}}_{\mathrm{it}}={{\alpha}_{1\mathrm i}\mathrm{lnCO}2\mathrm{PC}}_{\mathrm{it}-1}+{{\alpha}_{2\mathrm i}\mathrm{lnCO}2\mathrm{PC}}_{\mathrm{it}-2}+\beta_0+\beta_{1\mathrm i}\mathrm{lnRGDP}+\beta_{2\mathrm i}\mathrm{lnRDGP}2+\beta_{3\mathrm i}\mathrm{lnAIR}+\beta_{4\mathrm i}\mathrm{ECI}+\beta_{5\mathrm i}\mathrm{lnENU}+\beta_{6\mathrm i}\mathrm{lnEPU}{+\mathrm{lnENUECI}\beta_{7\mathrm i}+\mathrm{lnENUECI}\beta_{8i}+\mathrm u}_{\mathrm i,\mathrm t}{+\varepsilon}_{\mathrm i,\mathrm t}$$

## Results and discussion

### Pre-estimation diagnostics: descriptive statistics and correlation

To analyse fit model for a particular study, it is recommended to explore the main features of the data to see whether the variables present are symmetrical and have outliers, and to perform the necessary transformation on the variables. In this regard, this section presents the exploratory data analysis of the studied variables. It presents the summary of the descriptive statistics, correlation matrix and visualisation of the relationship, using a scatter plot, between the dependent and independent variables.

Table 3 unveils the summary statistics of the studied variables at logged (upper part) and at level (lower part). Based on the logged (i.e., logarithmic transformation value), the mean of log carbon emission per capita is 1.00 metric tonnes with a deviation of 1.38 metric tonnes. Log of real GDP (RGDP) and log of squared real GDP per capita (RGDP2) growth has a mean of $8.69 million and 77.54 million dollars, respectively, with a standard deviation of $1.43 million and $25.05 million respectively confirming that the degree of dispersion in the log of real GDP per capita is very low compared to dispersion in the log of squared real GDP per capita growth; that is, the range value of the latter is higher than the former as evidenced from the minimum and maximum value of the two economic variables. Furthermore, the log of air transport has 14.93 (*put units*), the standard deviation of 2.04 (*put units*), minimum of 8.85 (*put units*), and a maximum of 20.53 (*put units*). In the same way, the log of energy use has an average value of 4.81 (*put units*), the standard deviation of 0.52 (*put units*), a minimum of 3.66 (*put units*) and a maximum of 6.67 (*put units*).

**Table 3:** Summary statistics

Variable	Obs	Mean	Std. dev	Min	Max
Logged values					
LCO2PC	2,285	1.004414	1.382296	− 4.1178	4.249098
LRGDP	2,282	8.687264	1.439141	5.23387	11.42481
LRGDP2	2,282	77.53878	25.05686	27.3934	130.5263
LAIR	2,133	14.93206	2.044491	8.86376	20.52973
LENU	2,090	4.817426	0.527628	3.66612	6.675196
EPU	2,289	0.051619	0.044817	0	0.3267366
ECI	2,281	0.127056	0.988403	− 2.7911	2.8951
Variables at level					
CO_2_ emissions	2,285	5.528826	6.946781	0.01628	70.04223
GDP per capita	2,282	14,509.36	18,660.72	187.517	91,565.73
Air transport	2,141	20,900,000	73,900,000	0	824,000,000
Energy use	2,090	146.0758	108.6618	39.0998	792.5025
EPU	2,289	0.051619	0.044817	0	0.3267366
ECI	2,281	0.127056	0.988403	− 2.7911	2.8951

Regarding the summary statistics of variables at level, the mean of carbon emission per capita is 5.52 with the dispersion of 6.95 which is more than when it is at transformation level. Similarly, real GDP per capita growth, the standard deviation of $18 million, confirms that the degree of dispersion in the log of real GDP per capita is very low compared to dispersion in the level. Moreover, air transport emission has a mean and standard deviation of 209 (*put units*) and 739 (*put units*) respectively. Also, energy use has an average value of 146.08 (*put units*), the standard deviation of 108.66 (*put units*), a minimum of 39.10 (*put units*) and a maximum of 792.50 (*put units*). Finally, economic policy uncertainty and economic complexity have an average of 0.05 and 0.12, respectively, and dispersion of 0.04 and 0.10 respectively which means that economic policy uncertainty has fewer variations compared to the economic complexity index.

It should be noted that the observed summary statistics (variables at level) is more reliant than then logarithmic values; however, to avoid spurious results, logarithmic variables were employed in the model; hence, its exploratory statistics is also essential and that was why it was presented.

**Fig. 2:**
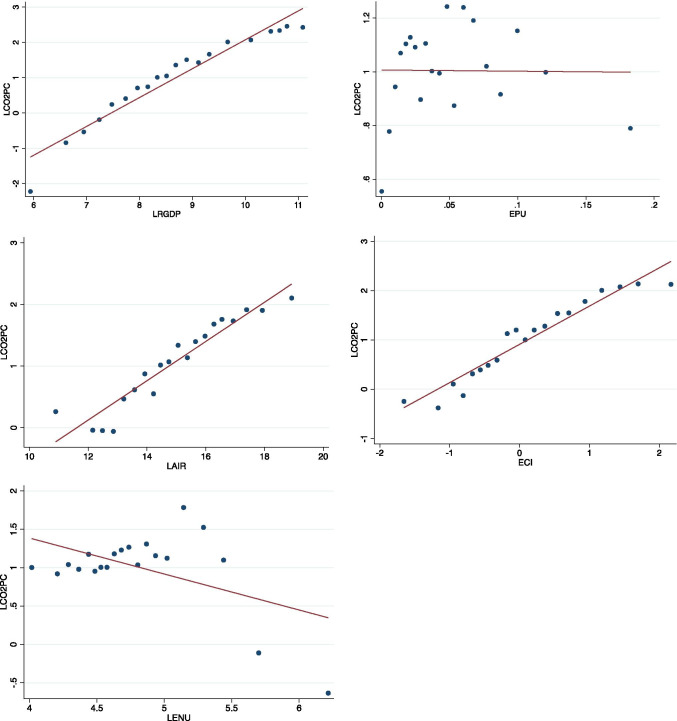
Scatter plots for variables of interest

Aside from the exploration of descriptive statistics, correlation matrix (Table 4) and scatter diagram (Fig. 2) were employed to find the pre-relationship between the log of dependent and log of independent variables. Both measure the same thing, just that whereas the correlation matrix shows the correlation coefficients and whether it is significant or not, the scatter diagram shows the visualisation, by using data points, of the relationship between the two variables with the fitted line superimposed on it. Starting from the correlation matrix in Table 4, it revealed that log of RGDP, RGDP2, AIR, and ECI has a positive and significant association with carbon emission per capita (CO2PC), i.e., as these variables and CO2PC increase with the same time as the carbon emission per capita. More importantly, the coefficients of RGDP and RGDP2 with CO2PC are 0.8532 and 0.8296 respectively meaning that the association between CO2PC and these variables is very strong and positive, thereby explaining why their observations with CO2PC are closely packed around the fitted line (Fig. 2). Also, the association between AIR and ECI with CO2PC is positive but while that of AIR with CO2PC is weakly positive, ECI with CO2PC is averagely positive, and the result was also asserted from the fitted line in the scatter plot (Fig. 2). On the opposite, ENU and EPU have a negative relationship with CO2PC. More importantly, ENU and CO2PC are very weak and significant while EPU and CO2PC are almost zero correlation but insignificant. This is also evidenced by the scatter plot in Fig. 2. The trend line in the plot of EPU and CO2PC is horizontally depicting a near-zero relationship.

**Table 4:** Correlation matrix

	LCO2PC	LRGDP	LRGDP2	LAIR	LENU	EPU	ECI
LCO2PC	1						
LRGDP	0.8532*	1					
	0.0000						
LRGDP2	0.8296*	0.9962*	1				
	0.0000	0.0000					
LAIR	0.4860*	0.5608*	0.5714*	1			
	0.0000	0.0000	0.0000				
LENU	− 0.1780*	− 0.4260*	− 0.4001*	− 0.2437*	1		
	0.0000	0.0000	0.0000	0.0000			
EPU	− 0.0015	0.0023	0.0005	0.0384	− 0.0055	1	
	0.9435	0.9127	0.9792	0.0759	0.802		
ECI	0.5543*	0.6753*	0.6787*	0.5081*	− 0.2905*	− 0.0262	1
	0.0000	0.0000	0.0000	0.0000	0.0000	0.2117	

^*^Correlation is significant at 0.05% (*p* < 0.05)

### Estimation of main results

After the exploratory data analysis of variable of interest has been performed, the section presents the panel estimation of the model with moderation variables (Table 5), and without moderation variable, but across the World Bank income clusters (Tables 6 and 7).

**Table 5:** Results for model

Variables (dep. variable: CO2PC, log)	Pooled OLS	Fixed effects	Random effects	System GMM	System GMM
Lagged LCO2PC				0.955***	0.955***
				(0.0455)	(0.0461)
LRGDP	3.375***	2.562***	2.593***	0.301***	0.284***
	(0.133)	(0.347)	(0.339)	(0.0747)	(0.0724)
LRGDP2	− 0.145***	− 0.0937***	− 0.0958***	− 0.0141***	− 0.0129***
	(0.00736)	(0.0191)	(0.0186)	(0.00356)	(0.00350)
LAIR	0.0546***	0.0184*	0.0195*	0.00611***	0.00628***
	(0.00706)	(0.0104)	(0.0100)	(0.00230)	(0.00230)
LENU	0.989***	0.874***	0.876***	0.0604***	0.0687***
	(0.0223)	(0.141)	(0.132)	(0.0154)	(0.0160)
EPU	− 0.0745	− 0.0296	− 0.0289	0.0114	0.270
	(0.271)	(0.0861)	(0.0858)	(0.0603)	(0.561)
ECI	− 0.00252	0.0354	0.0335	− 0.00887*	− 0.0977**
	(0.0161)	(0.0268)	(0.0263)	(0.00461)	(0.0380)
LENUECI					0.0186**
					(0.00768)
LENUEPU					− 0.0521
					(0.121)
Lower middle income	1.093***		1.203***		
	(0.1000)		(0.301)		
Upper middle income	1.094***		1.205***		
	(0.109)		(0.303)		
High income	1.055***		0.894**		
	(0.118)		(0.365)		
Constant	− 23.59***	− 18.45***	− 19.63***	− 1.817***	− 1.796***
	(0.580)	(2.044)	(1.923)	(0.435)	(0.416)
Year dummies	Yes	Yes	Yes		
AR (2) *p*-value				0.934	0.916
Hausman (*p*-value)		0.0000			
Hausman *χ*^2^(6)		46.39			
Observations	1,935	1,935	1,935	1,734	1,734
*R*-squared	0.886	0.618			
Number of country ID		109	109	109	109

Robust standard errors in parentheses. ***p < 0.01, **p < 0.05 and *p < 0.1 represent 1%, 5% and 10% levels of significance respectively. Low income is used as the reference group

**Table 6:** Pooled OLS (or LSDV)

Variables (dependent variable: LCO2PC)	Low income	Lower middle income	Upper middle income	High income
LRGDP	1.656	0.142	5.966***	3.917***
	(3.426)	(0.823)	(0.607)	(0.516)
LRGDP2	− 0.0748	0.0679	− 0.301***	− 0.165***
	(0.288)	(0.0580)	(0.0371)	(0.0260)
LAIR	0.104	0.113***	0.00392	0.0276***
	(0.109)	(0.0200)	(0.00661)	(0.00660)
LENU	− 0.515	0.869***	1.149***	0.856***
	(0.336)	(0.0593)	(0.0217)	(0.0338)
EPU	− 0.172	− 0.966*	0.787**	0.388
	(1.398)	(0.578)	(0.349)	(0.304)
ECI	0.983***	0.369***	0.0870***	− 0.204***
	(0.214)	(0.0474)	(0.0184)	(0.0134)
Constant	− 6.506	− 10.26***	− 33.35***	− 24.81***
	(11.40)	(2.908)	(2.465)	(2.532)
Year dummies	Yes	Yes	Yes	Yes
Observations	92	485	655	703
R-squared	0.700	0.664	0.826	0.773

Robust standard errors in parentheses. ***p < 0.01, **p < 0.05 and *p < 0.1 represents 1%, 5% and 10% levels of significance respectively

**Table 7:** Fixed and random effects estimates

Variables (dep. variable: CO2PC, log)	Low income	Lower middle income	Upper middle income	High income	Low income	Lower middle income	Upper middle income	High income
	Fixed effects	Random effects						
LRGDP	4.542**	0.573	1.811*	2.421***	1.656	0.564	1.870*	2.811***
	(1.740)	(1.146)	(1.031)	(0.558)	(6.039)	(1.152)	(1.029)	(0.525)
LRGDP2	− 0.176	0.0711	− 0.0517	− 0.0749**	− 0.0748	0.0677	− 0.0538	− 0.0987***
	(0.132)	(0.0856)	(0.0598)	(0.0289)	(0.515)	(0.0861)	(0.0599)	(0.0263)
LAIR	− 0.0471	− 0.0393	0.0528***	0.00694	0.104	− 0.0255	0.0483***	0.00298
	(0.0676)	(0.0306)	(0.0177)	(0.0116)	(0.220)	(0.0293)	(0.0166)	(0.0107)
LENU	2.122***	1.056***	0.662***	0.949***	− 0.515	1.022***	0.703***	0.904***
	(0.407)	(0.168)	(0.211)	(0.0809)	(0.959)	(0.157)	(0.201)	(0.0747)
EPU	− 0.401	0.147	0.00751	− 0.0614	− 0.172	0.151	− 0.00938	− 0.0611
	(0.384)	(0.225)	(0.105)	(0.101)	(1.315)	(0.224)	(0.104)	(0.0987)
ECI	0.0152	0.150*	0.0272	− 0.0342	0.983**	0.157**	0.0242	− 0.0532**
	(0.0504)	(0.0778)	(0.0357)	(0.0303)	(0.383)	(0.0786)	(0.0332)	(0.0268)
Constant	− 34.92***	− 12.52***	− 14.37***	− 19.18***	− 6.506	− 12.31***	− 14.84***	− 20.40***
	(8.232)	(4.279)	(5.148)	(2.787)	(22.34)	(4.225)	(5.085)	(2.693)
Year dummies	Yes	Yes	Yes	Yes	Yes	Yes	Yes	Yes
Observations	92	485	655	703	92	485	655	703
*R*-squared	0.859	0.673	0.716	0.602				
Number of country ID	6	28	37	38	6	28	37	38

Robust standard errors in parentheses. ***p < 0.01, **p < 0.05 and *p < 0.1 represents 1%, 5% and 10% levels of significance respectively

The first second column is the estimation of the result from pooled OLS; it shows that real GDP per capita and squared real GDP per capita growth have a positive and negative relationship with carbon emission at a 1% level of significance. Put clearly, a unit rise in real GDP per capita contributes to a 3.375% rise in carbon emission, and a unit rise in square GDP per capita reduces carbon emission by 0.145%. What this result justifies is that increment of GDP to a certain point is a perfect measure to diminish the carbon emission, hence validating the existence of the EKC hypothesis. Another significant predictor of carbon emission under this model is air transport and energy use because both have a positive and significant relationship at the 1% level; in fact, additional use of air transport systems and energy use releases about 0.05% and 0.99% increase in carbon emission. Using lower income as benchmark cluster, carbon emission that emanates from lower middle income, upper middle income, and high-income countries are 1.093% times, 1.094% times, and 1.055% times more emission that emanates from the lower income country. R-squared value of 0.886 in this model supports 88.6% of the variation in carbon emission that was explained by significant independent variables.

Based on the fixed effect (FE) and random effect (FE) model estimation, the number of significant independent variables was the same as the one under pooled OLS estimation, and their coefficients were noticed to have decreased. For instance, the coefficient of real GDP per capita growth implies a 2.56% and 2.59% increase in carbon emission under FE and RE model respectively. The coefficient of squared GDP per capita gives carbon emission 0.093% and 0.096% reduction, thus also confirming the existence of the EKC hypothesis as explained under pooled OLS. Air transport, at a 10% level, also increases emission by 0.02%, while energy use increases it by 0.87% at a 1% level of significance. Furthermore, the income clusters under the random model support that more carbon emission is released in lower middle-income, upper middle-income and high-income countries.

For robustness check, system GMM estimation, which shows the relationship lagged dependent variable on independent variables to eliminate the serial correlation in the model, was employed. The last two columns in Table 5 show the result of the model with moderating variables and without moderating variables. In the result, the EKC hypothesis was also significantly confirmed at a 1% level of significance, because both real GDP and squared GDP positively and negatively affect the carbon emission per capita. That is, while real GDP fosters the emission by 0.284–0.301%, it is squared to decrease it by 0.01–0.02%; that is, as economic growth skyrockets, carbon emission would begin to reduce. The same result was confirmed with air transport and energy use because both variables with(out) moderating variables significantly increase carbon emission. Additionally, the system GMM model revealed that the economic complexity index significantly reduces carbon emission by 0.01–0.09% while economic policy uncertainty, just like in other panel model techniques, does not significantly relate to carbon emission. Furthermore, the coefficient of interaction variables (LENUECI) and (LENUEPU) were also taken care of. However, while ECI significantly moderates energy use by reducing its effects on carbon emission by 0.02%, EPU is not a significant moderator even though it has a negative impact on carbon emission. In addition, the diagnostic measures support no serial correlation in the error term, AR(*p*) > 0.05.

Next is the estimation of carbon emission in World Bank income clusters. Three-panel techniques used here are pooled OLS (Table 6), FE and RE model (Table 7). The interpretation of the result is followed thus. In Table 6, which revealed the pooled OLS technique of the income clusters, all the predictors under the low-income cluster did not significantly predict LCO2PC except ECI which had a positive and significant relationship with LCO2PC—rise in ECI upsurge CO2PC by 0.98%. Under lower middle-income clusters, air transport and energy use significantly predict CO_2_ by 0.11% increase and 0.87% at 1% level increase respectively. Also, while energy use reduces the emission by a 0.97% decrease, ECI upsurges it with a 0.37% increase. In the case of an upper middle-income country, only air transport has no significant relationship with CO_2_. Real GDP increases the emission by 5.97%, while RGDP squared reduces it by 0.30% thereby confirming the EKC hypothesis; energy use emanates it by 1.15%, EPU increases it by 0.78% and ECI increases it by 0.09%. For high-income country, all the predictors except EPU significantly predict the CO2PC; the EKC hypothesis is also confirmed under this cluster, and air transport and energy use have a positive impact by fostering the emission by 0.03% and 0.86% respectively, whereas ECI in this cluster reduces the emission by 0.20% decrease. The R-squared value across the income clusters is 66.4–82.6% indicating a large percentage of variation in CO_2_ which was explained by the significant predictors in the model.

Furthermore, in Table 7, we have the FE and RE estimation for comparative analysis among the four World Bank income clusters. The FE estimation of income clusters showed that only real GDP (by 4.54% increase) and energy use (by 2.12% increase) are a significant predictor of CO2PC under low-income countries, and only energy use with a 1.05% increase is a significant predictor of CO2PC under low middle-income countries. However, under upper middle-income countries, real GDP per capita, air transport, and energy have positive and significant impacts on CO2PC. Favourably, there is the existence of the EKC hypothesis in the high-income country as real GDP and squared real GDP has a significant positive and negative effect on CO2PC. Regarding RE estimation of the clusters, only ECI (by 0.98% increase) significantly predicts CO2PC in low-income countries; in lower middle-income clusters, energy use and ECI significantly predict CO2PC by 1.02% increase and 0.16% increase respectively. For upper middle-income clusters, an increased surge in real GDP, air transport, and energy was predicted to significantly surge CO2PC 1.87%, 0.05%, and 0.70% respectively. Like other models, the ECK hypothesis was also established in high-income clusters under RE with the addition of a decrease in CO2PC by 0.05% resulting from an improvement ECI.

## Conclusion and policy implications

Climate change is an inevitable and urgent global challenge with long-term implications for the sustainable economic performance of all countries. It is a burning issue that has been addressing by researchers in an environmental and related field. Therefore, this study examines the determinants of carbon emissions, and special interest is given to the level of economic complexity and economic policy uncertainty as moderating variables. This study used World Bank income clusters from 1996 to 2016 as the target population, and panel model estimation was employed to carve out a comparative analysis of the clusters while also grouping the clusters as the main model. Based on the main model (grouped clusters) estimations, the result revealed the existence of the EKC hypothesis.

The results indeed support a view of researchers such as Can and Gozgor (2017), Neagu (2019), Chu (2020) and Satrovic and Ahmad (2021). This means that as economic growth and carbon emission increase, it gets to a certain threshold carbon emission decline while observing a continuous increase in economic growth. Also, an increase in the air transport system and high consumption of energy release more carbon emissions to the climate. In addition, improvement in the productive capacity of the large economic system (otherwise known as ECI) decreases carbon emission significantly. This is contrary to the study of Kaufmann et al. (1998); Mealy et al. (2019) oppose it saying that ECI increases emission in their study, but the result was in line with the study of Boleti et al. (2021) who find that ECI has a negative impact on environmental emission. Economic policy uncertainties (EPUs) do not have a significant impact.

Moreover, the study revealed that ECI moderated the impact of other variables on emission, but EPU is not a significant moderator. Furthermore, comparative analyses among the four income clusters, like the study of Doğan et al. (2019), were made. The outcome of the result confirmed an EKC hypothesis only in the high-income clusters; ECI is a significant predictor of carbon emission in the four clusters, but it only decreases the emission in high-income clusters asserting the productive capacity of the high-income clusters. Based on the conclusion, the following policy implication was recommended. The necessity to improve the economic growth cannot be jeopardised at the expense of disappearing the environmental change, so policymakers need ways to synergize on energy conservation strategy especially in each income cluster; also since ECI moderates the relationship between the carbon emission and the independent variable of interest, a direction for policy is to ensure that economic complexity is considered in the process of defining macroeconomic and energy conservation policies.

## Data Availability

The datasets generated and/or analysed during the current study are not publicly available but are available from the corresponding author on reasonable request.

## Appendix

**Table 8:** List of countries in sample

1	2	3	4
**Low income**	**Lower middle income**	**Upper middle income**	**High income**
Congo, Dem. Rep	Angola	Albania	Australia
Ethiopia	Bangladesh	Algeria	Austria
Mozambique	Bolivia	Argentina	Belgium
Tajikistan	Cambodia	Armenia	Canada
Tanzania	Cameroon	Azerbaijan	Chile
Togo	Egypt, Arab Rep	Belarus	Croatia
	El Salvador	Bosnia and Herzegovina	Czech Republic
	Ghana	Botswana	Denmark
	Honduras	Brazil	Finland
	India	Bulgaria	France
	Indonesia	China	Germany
	Kenya	Colombia	Greece
	Kyrgyz Republic	Costa Rica	Hungary
	Moldova	Dominican Republic	Ireland
	Mongolia	Ecuador	Israel
	Morocco	Gabon	Italy
	Myanmar	Georgia	Japan
	Nicaragua	Guatemala	Kuwait
	Nigeria	Iran, Islamic Rep	Latvia
	Pakistan	Jamaica	Lithuania
	Philippines	Jordan	Netherlands
	Senegal	Kazakhstan	New Zealand
	Tunisia	Lebanon	Norway
	Ukraine	Libya	Oman
	Uzbekistan	Malaysia	Panama
	Vietnam	Mexico	Poland
	Zambia	Namibia	Portugal
	Zimbabwe	North Macedonia	Qatar
		Paraguay	Saudi Arabia
		Peru	Singapore
		Romania	Slovak Republic
		Russian Federation	Slovenia
		South Africa	Spain
		Sri Lanka	Sweden
		Thailand	Switzerland
		Turkey	United Arab Emirates
		Turkmenistan	UK
			USE
